# Navigating the potential and pitfalls of large language models in patient-centered medication guidance and self-decision support

**DOI:** 10.3389/fmed.2025.1527864

**Published:** 2025-01-23

**Authors:** Serhat Aydin, Mert Karabacak, Victoria Vlachos, Konstantinos Margetis

**Affiliations:** ^1^School of Medicine, Koç University, Istanbul, Türkiye; ^2^Department of Neurosurgery, Mount Sinai Health System, New York, NY, United States; ^3^College of Human Ecology, Cornell University, Ithaca, NY, United States

**Keywords:** Large Language Models, ChatGPT, patient education, self-medication, artificial intelligence, machine learning, deep learning

## Abstract

Large Language Models (LLMs) are transforming patient education in medication management by providing accessible information to support healthcare decision-making. Building on our recent scoping review of LLMs in patient education, this perspective examines their specific role in medication guidance. These artificial intelligence (AI)-driven tools can generate comprehensive responses about drug interactions, side effects, and emergency care protocols, potentially enhancing patient autonomy in medication decisions. However, significant challenges exist, including the risk of misinformation and the complexity of providing accurate drug information without access to individual patient data. Safety concerns are particularly acute when patients rely solely on AI-generated advice for self-medication decisions. This perspective analyzes current capabilities, examines critical limitations, and raises questions regarding the possible integration of LLMs in medication guidance. We emphasize the need for regulatory oversight to ensure these tools serve as supplements to, rather than replacements for, professional healthcare guidance.

## KEY ASPECTS

• LLMs are transforming patient education by offering easily accessible and user-friendly guidance on medication use, improving patient understanding and self-management.
• These models may empower patients in remote or underserved areas by providing immediate, reliable information on health conditions and self-care, especially where healthcare access is limited.
• However, challenges remain in ensuring accuracy, particularly in complex cases due to the current limitations in accessing real-time data and personalized patient information.
• There are ethical concerns regarding the use of LLMs for self-medication guidance without healthcare oversight, which may lead to unintended health risks.
• To improve safety, future efforts should focus on integrating real-time medical databases and establishing clear regulations for the use of LLMs in healthcare contexts.

## 1 Introduction

The Large Language Models (LLMs) represent a significant advancement in patient education, particularly in personalized health and medication counseling. Leading examples such as OpenAI’s ChatGPT (1), and Google’s Gemini (2) can process extensive datasets and engage in conversational interactions. These artificial intelligence (AI) applications are increasingly being explored in healthcare to provide drug information, help patients navigate complex medication regimens, and guide initial responses to medical situations. By generating information of variable reliability, the extent to which LLMs can effectively influence patient autonomy in self-medication decisions and healthcare choices remains an open question.

The appeal of LLMs in healthcare stems from their accessibility and ease of use. Patients can readily access information about medication dosages, interactions, side effects, and alternatives without waiting to consult a healthcare provider. These models can enhance health literacy by translating medical jargon into plain language, helping patients make informed decisions about over-the-counter medications and some prescribed treatments. For example, studies show that LLMs can provide basic guidance for immediate-response situations, such as initial management of snakebites or other common conditions requiring urgent attention (3).

However, significant challenges exist in safely integrating LLMs into patient self-care decisions. A primary concern is the reliability of LLM-generated information, particularly regarding complex drug interactions or rare conditions. Cases of AI systems providing incorrect or misleading information have been documented, notably in sensitive areas with significant health and ethical implications, such as self-managed medication abortion (4).

Building upon our recent scoping review that identified six major themes in LLM applications for patient education (5), this article examines one critical theme: the role of LLMs in patient-centered medication guidance and self-decision support. We assess both the potential of LLMs to enhance autonomous medication use and the risks associated with their misuse or misunderstanding. This perspective article reviews recent advances, identifies key challenges, and proposes future directions for LLM implementation that balance patient autonomy with healthcare safety and ethical standards. By examining this specific theme in detail, we aim to contribute targeted insights into the responsible integration of LLM technology in medication guidance while addressing critical questions about patient safety and ethical implementation.

## 2 Current advances in LLMs for customized medication use and self-decision

### 2.1 LLMs as informational aids for drug interactions and side effects

LLMs show promise as informational resources for medication guidance, particularly in explaining drug interactions, potential side effects, and usage instructions. These models can translate complex pharmacological information into accessible language for patients with limited medical knowledge. This capability helps patients better understand their medication regimens and may reduce drug-drug interactions caused by misunderstandings (6, 7).

A recent study by Iqbal et al. examined ChatGPT’s reliability as a secondary opinion source for dermatological treatments (8). While dermatologists approved 98.87% of the model’s medication suggestions, they identified limitations such as incorrect Anatomical Therapeutic Chemical codes and errors in drug route specifications. These findings suggest that while ChatGPT shows promise for general treatment guidance, it requires further refinement for precise clinical applications.

LLMs also demonstrate potential in helping patients manage complex medication regimens, particularly in cases of polypharmacy where drug-drug interactions pose significant risks. Research shows that these models can effectively identify and explain risks associated with specific drug combinations, including interactions between over-the-counter medications and treatments for chronic conditions (9). This capability could help prevent medication errors and resulting hospitalizations from adverse drug reactions.

Recent research also explores LLMs’ potential in helping healthcare professionals screen for drug interactions. A comparative analysis of ChatGPT, Google Bard, and Bing AI found that while these tools do not yet match the accuracy of specialized clinical software, they can effectively identify relevant drug interactions in real-time. Among the tested models, Bing AI demonstrated the highest accuracy and specificity, while ChatGPT-4 showed improvements over its predecessor (6). These findings highlight the need for further development of LLM capabilities, indicating that while they show potential, they are not yet ready for reliable use in clinical settings but may be in the future.

### 2.2 Facilitating self-decision in self-administered treatments

LLMs show potential in guiding patients through self-administered treatments, particularly in situations requiring immediate action. For example, studies have evaluated ChatGPT’s ability to provide first-aid advice for venomous snakebites while emphasizing the need for urgent medical care (3). This capability could be particularly valuable in remote areas with limited healthcare access, offering patients guidance to take appropriate immediate actions while awaiting professional care. Infrastructural challenges, such as unreliable internet connectivity, may hinder its implementation in such settings, though its potential remains promising. However, researchers found that while ChatGPT-3.5 provided reliable general guidance, it should not replace professional medical consultation, especially in critical situations. The study emphasized the need for continued improvements to enhance AI’s reliability in high-stakes medical scenarios.

Roosan et al. evaluated ChatGPT’s effectiveness in Medication Therapy Management, focusing on drug interaction identification and therapeutic adjustments (10). While ChatGPT-4 demonstrated high accuracy with simple and moderately complex cases, it showed limitations when handling complex scenarios requiring patient-specific considerations. The model proved capable of identifying common drug-drug interactions but struggled with personalized dosage adjustments, highlighting the continued need for human oversight in clinical decision-making.

## 3 Challenges and limitations in LLMs for medication guidance and self-decision

### 3.1 Inaccuracy and misleading information

A critical challenge in using LLMs for medication guidance is their potential to generate inaccurate or misleading information. While these models can process large datasets, they lack access to real-time, continuously updated medical databases, potentially leading to outdated or incorrect advice. For example, studies have found that ChatGPT-3.5 provided inaccurate information about self-managed medication abortion, exaggerating risks despite evidence supporting its safety when properly administered (4). Such misinformation can increase patient anxiety, perpetuate stigma, and discourage evidence-based healthcare decisions.

Research by Sheikh et al. compared ChatGPT-3.5 and ChatGPT-4’s ability to assess the safety of non-prescription medications and supplements for patients with kidney disease (11). While ChatGPT-4 showed improvement over its predecessor (81.4% vs 64.5% concordance with Micromedex), neither matched the reliability of established drug information resources. Both models particularly struggled with supplement safety assessments, often defaulting to “unknown toxicity” classifications due to limited data.

Rao et al. (9) assessed ChatGPT-3.5’s role in managing polypharmacy in geriatric patients, finding its deprescribing recommendations aligned with guidelines for patients without cardiovascular disease but lacked accuracy when factoring in functional impairments and cardiovascular history. Notably, it often recommended deprescribing pain medications without considering older adults’ pain management needs. Similarly, in cases of renal dysfunction, ChatGPT achieved only 16.7% accuracy in dose adjustments incorporating patient-specific variables such as renal markers and comorbidities (12). These findings highlight the limitations of LLMs in complex scenarios requiring personalized clinical expertise, emphasizing their role as supplementary tools rather than replacements for professional judgment. This low accuracy poses significant risks in clinical settings where precise dosing is crucial, demonstrating that while LLMs may support preliminary decision-making, they cannot reliably replace clinical expertise in complex medical situations.

### 3.2 Ethical and safety concerns in self-decision support

The use of LLMs for self-medication guidance raises significant ethical concerns, particularly when patients use these tools without healthcare professional oversight. A primary risk is that LLMs may provide seemingly authoritative advice that lacks clinical nuance, potentially encouraging unsafe medical decisions. This risk is heightened in regions with limited healthcare access, where patients might rely on AI as their primary medical information source.

Hsu et al. examined ChatGPT’s ability to handle medication consultations and drug-herb interaction questions (13). While the model effectively addressed basic public inquiries, it performed poorly on complex questions from healthcare providers. The study revealed particular limitations in analyzing interactions between traditional Chinese and Western medicines, often providing vague or incomplete information. These findings indicate that while ChatGPT can help with basic medication questions, it currently lacks the sophistication needed for reliable guidance in specialized clinical contexts.

Ethical concerns also emerge in managing sensitive medical conditions, such as cancer. When evaluated for cancer symptom management guidance, ChatGPT’s recommendations showed notable discrepancies from National Comprehensive Cancer Network (NCCN) guidelines. The model tended to provide generalized advice that failed to address the complex symptom burdens typical of cancer patients (14). This gap between AI-generated recommendations and evidence-based guidelines underscores the risks of relying on LLMs for critical health decisions.

Privacy constraints prevent LLMs from accessing individual medical records, limiting their ability to provide personalized recommendations. This limitation is particularly problematic for high-risk populations, including elderly patients and those with chronic illnesses, who require carefully tailored treatment plans. Without access to patient-specific data, LLMs default to generalized advice that may be inappropriate or unsafe for complex medical conditions. As demonstrated in previous research, ChatGPT’s inability to consider specific renal function metrics led to incorrect dosing recommendations for patients with kidney disease, illustrating the potential safety risks of such limitations (12).

These limitations highlight the critical need for a structured ethical framework governing LLM deployment in healthcare. The integration of AI into patient self-decision support requires a balanced approach that positions these tools as supplements to, not replacements for, professional medical expertise. A collaborative model combining AI capabilities with clinical oversight could optimize the benefits of LLMs while minimizing risks. The development of robust regulatory guidelines will be essential to harness LLM potential while maintaining patient safety and ethical standards.

## 4 Future directions and recommendations

### 4.1 Improving accuracy and reliability of LLMs for medication-related information

Enhancing LLM reliability for medication guidance requires integration with real-time medical databases and continuous content updates. Connecting these models to current pharmacological databases would enable access to the latest drug interaction guidelines, side effect profiles, and dosage recommendations. Such integration could help align AI systems with evolving healthcare information while improving response accuracy for patient inquiries. Development of frameworks allowing LLMs to access validated sources such as PubMed, FDA databases, and regional repositories would strengthen the clinical relevance of their recommendations.

Specialized training protocols represent another key avenue for improvement, particularly in enhancing LLMs’ contextual understanding of patient inquiries. Targeted training in medical ethics and patient safety could reduce risks in high-stakes areas such as mental health, reproductive health, and complex medication management. Collaboration between healthcare professionals and AI developers is crucial for ensuring these models meet clinical standards. By involving medical experts in model refinement, especially for context-specific information and decision-making guidance, developers can better align AI outputs with the nuanced requirements of personalized medicine. Strategic partnerships between AI companies and medical institutions could facilitate ongoing model validation and improvement.

### 4.2 Balancing autonomy with safety: ethical and regulatory perspectives

The growing role of LLMs in medication guidance necessitates an ethical framework balancing patient autonomy with safety. Our previous scoping review highlighted that while LLMs effectively simplify medical terminology, they often lack reliability in critical, high-stakes scenarios (5). This finding underscores the need for comprehensive regulatory standards ensuring transparency in AI recommendations, including clear disclaimers about the importance of professional medical consultation. Such guidelines would help users understand that AI-generated advice supplements, rather than replaces, clinical expertise.

Looking forward, establishing medical AI ethical review boards, similar to institutional review boards for clinical research, could provide structured oversight of LLM implementation. These boards could evaluate training data, assess response biases, and monitor AI applications in patient education and self-care. This framework would ensure AI development aligns with patient safety priorities and evolving healthcare policies.

## 5 Discussion

LLMs show promise in supporting patient self-decision making for medication use, providing accessible, on-demand resources for drug-related information. These tools help patients explore questions about drug interactions, side effects, and medication schedules, potentially enhancing health literacy and informed decision-making. However, significant limitations and risks exist. The inability of LLMs to incorporate individual patient data, including medical histories and current medications, creates a fundamental barrier to personalized advice. this limitation, combined with potential inaccuracies in AI-generated responses, necessitates careful integration of LLMs into healthcare, particularly in sensitive areas such as reproductive and mental health.

In environments where access to healthcare professionals is limited or communication systems are disrupted, such as remote areas or disaster zones, LLMs can provide support for patient self-care. These AI tools can deliver immediate, situation-specific advice for managing medical concerns when professional help is unavailable. This immediate guidance can be life-saving in cases where there are no healthcare facilities nearby, offering a sense of empowerment and structured steps for non-professionals facing medical emergencies. Nevertheless, while LLMs can provide a valuable bridge until medical assistance is available, they cannot replace the expertise of healthcare professionals in complex or high-stakes situations. As such, their recommendations should emphasize the provisional nature of AI guidance in austere environments, ideally directing individuals to seek professional care as soon as circumstances allow.

In addition to emergencies, LLMs can be used to support patients in everyday medication decisions, particularly with over-the-counter (OTC) drugs. Many individuals may not fully understand the risks of combining OTC medications with prescription drugs or specific medical conditions, often due to the complex and lengthy drug information provided on packaging. Patients may also assume OTC medications are inherently safe or may avoid consulting healthcare professionals for minor issues. In such cases, LLMs can assist by analyzing drug information and identifying potential interactions or contraindications based on a patient’s reported medications and medical conditions. This guidance can help patients make safer choices, promoting informed self-care in routine health decisions. However, the accuracy and safety of these recommendations depend on LLMs being continuously updated with the latest clinical data. The potential for adverse outcomes highlights the need for rigorous oversight, ensuring that LLM-driven advice is a safe, supplementary resource in patient-centered healthcare.

While LLMs can empower patients with information, the risks of misinformation or oversimplified guidance are substantial, especially if patients bypass professional medical consultation in favor of AI recommendations. Future developments must address both accuracy and ethical considerations. Key improvements should include integrating validated medical databases and increased collaboration with healthcare professionals. Additionally, regulatory oversight must establish clear boundaries for LLM use, ensuring these tools serve as supportive rather than standalone resources. Clear disclaimers and transparent communication about AI limitations can help position LLMs as supplements to professional healthcare guidance.

LLMs represent a transformative development in patient education, potentially reshaping how patients approach self-medication and health decisions. Their successful implementation depends on addressing current limitations in probabilistic data synthesis, personalization capabilities, and ethical considerations in sensitive healthcare areas. The path forward requires balancing AI’s informational capabilities with professional medical guidance while maintaining focus on patient safety and autonomy. This balanced approach will be crucial for realizing the full potential of LLMs in patient-centered healthcare.

## Data Availability

The original contributions presented in this study are included in this article/supplementary material, further inquiries can be directed to the corresponding author.
